# Prognostic factors and treatment impact on overall survival in patients with renal neuroendocrine tumour

**DOI:** 10.1002/bco2.341

**Published:** 2024-03-11

**Authors:** Olamide O. Omidele, Christopher Connors, Nikhil Wainganker, Ketan Badani, John Sfakianos, Reza Mehrazin, Isuru Jayaratna

**Affiliations:** ^1^ Department of Urology Icahn School of Medicine at Mount Sinai New York New York USA

**Keywords:** kidney tumours, neuroendocrine carcinoma, partial nephrectomy, radical nephrectomy, renal cancer

## Abstract

**Background:**

Renal neuroendocrine neoplasms (R‐NEN) are exceptionally rare tumours characterized by high mortality rates.

**Objective:**

The objective of this study is to analyse prognostic factors and treatment impact on overall survival in patients with R‐NEN.

**Design, setting and participants:**

We identified all patients with R‐NEN in the National Cancer Database (NCDB) from 2004 to 2019 and identified prognostic factors for improved survival.

**Results and limitations:**

Of 542 R‐NEN cases, 166 (31%) were neuroendocrine tumour grade 1 (NET‐G1), 14 (3%) were neuroendocrine tumour grade 2 (NET‐G2), 169 (31%) were neuroendocrine carcinoma (NEC‐NOS), 18 (3%) were large cell neuroendocrine carcinoma (LC‐NEC) and 175 (32%) were small cell neuroendocrine carcinoma (SC‐NEC). Median overall survival for all patients in the study was 44.88 months (SE, 4.265; 95% CI, 27.57–62.19). Median overall survival was 7.89 months (SE 0.67; 95% CI, 6.58–9.20) for patients without surgical intervention and 136.61 months (SE 16.44; 95% CI, 104.38–168.84, *p* < 0.001) for patients who underwent surgery. Increased age (HR, 1.05; 95% CI, 1.03–1.06; *p* < 0.001), T4 stage disease (HR, 3.17; 95% CI, 1.96–5.1; *p* < 0.001), NEC‐NOS histology (HR, 2.82; 95% CI, 1.64–4.86; *p* < 0.001), LC‐NEC histology (HR, 2.73; 95% CI, 1.04–7.17; *p* = 0.041) and SC‐NEC histology (HR, 5.17; 95% CI, 2.95–9.05; *p* < 0.001) were all positive predictors of worsening overall survival. The main limitation of the study is its retrospective design.

**Conclusion:**

R‐NEN is an aggressive tumour characterized by high mortality rates. Surgery continues to be the mainstay of treatment and has shown to provide a survival benefit for most patients.

**Patient Summary:**

R‐NEN is composed of several tumour histologies that differ based on their aggressiveness with NEC‐NOS and SC‐NEC being the most lethal. Surgery, predominantly through minimally invasive approaches, is the mainstay of treatment and has a clear survival benefit.

## 1 INTRODUCTION

Neuroendocrine neoplasms (NEN) are rare tumours with reported age‐adjusted incidence of 7 out of 100 000 individuals in the United States.^1^, ^2^ NENs are composed of tumours linked by histological markers that can arise in any organ, including those that do not harbour neuroendocrine cells.^3^ The most common primary sites for NENs are the gastrointestinal tract, lungs and the pancreas.^4^, ^5^ These tumours have been difficult to characterize due to their varying biologic behaviour, histology and treatment response.^3^, ^6^

Renal NENs (R‐NEN) are exceptionally rare, with reports of around 100 cases published in the literature.^3^, ^7^, ^8^, ^9^, ^10^ They can be broadly classified as either well‐differentiated neuroendocrine tumours (NET) or poorly differentiated neuroendocrine carcinomas (NEC).^11^ Renal NEC can further be subdivided into large cell neuroendocrine carcinomas (LC‐NEC) or small cell neuroendocrine carcinomas (SC‐NEC).^3^, ^6^

There is a paucity of data on primary R‐NEN with most of the current literature composed of small case series or individual case reports.^9^, ^12^ Thus, there is a need for population‐based data to understand treatment trends and overall survival for patients with R‐NEN in hopes of better characterizing which patients may benefit from specific treatments based on their underline histology. We present the largest population‐based study of patients with primary R‐NEN, characterize treatment options and provide predictors of worsening overall survival.

## 2 METHODS

### 2.1 Study population

The National Cancer Database (NCDB) was queried from 2004 to 2019 to identify adult patients with R‐NENs. The clinical oncology database consists of data collected from over 1500 accredited hospitals across the United States and reports approximately 72% of newly diagnosed cases in the country. Primary site code C64.9 (Kidney, NOS) was used to identify 668 400 cases of renal cancer. Histology codes 8240/3 neuroendocrine tumour, Grade 1 (NET‐G1), 8249/3 neuroendocrine tumour, Grade 2 (NET‐G2), 8246/3 neuroendocrine carcinoma, NOS (NEC‐NOS), 8013/3 large cell neuroendocrine carcinoma (LC‐NEC) and 8041/3 small cell neuroendocrine carcinoma (SC‐NEC) were used to identify 663 cases of R‐NEN. Patients under 18 years of age and those with a renal pelvis neoplasm were excluded, leaving 542 patients included in the study.

### 2.2 Variables studied

The following variables were extracted and analysed:

- Baseline characteristics: age at diagnosis, sex, race, Hispanic ethnicity, insurance status, rural or urban classification, treatment facility type and Charlson–Deyo score.
- Tumour characteristics: histological classification, tumour size, laterality and AJCC clinical stage at diagnosis, sites of metastasis.
- Treatment details: surgical intervention, treatment with chemotherapy, radiation or immunotherapy, type of surgery, timing of surgery, surgical approach and time from diagnosis to surgery.
- Outcomes: status of surgical margins, mean and median OS, 1‐, 3‐, 5‐ and 10‐year mortality.

### 2.3 Statistical analysis

Baseline characteristics were tabulated for all R‐NEN patients. A median and an interquartile range were calculated for continuous variables, while absolute numbers and proportions were reported for categorical variables. Kaplan–Meier analysis was used to estimate 1‐, 3‐, 5‐ and 10‐year OS estimates for R‐NEN patients along with mean and median OS. Survival times represented the months elapsed from diagnosis to death. Additional Kaplan–Meier survival curves were generated to compare survival outcomes based on overall AJCC stage at diagnosis, AJCC T stage at diagnosis, histologic subtype and the presence of surgical intervention. Log‐rank tests were used to detect significant differences in survival between groups. Case–control matching was used to compare overall mortality between patients diagnosed with R‐NEN and clear cell renal cell carcinoma (ccRCC). Patients with ccRCC were identified in the NCDB database using the histological code 8310/3. R‐NEN and ccRCC cohorts were matched on age, sex, AJCC T stage, and Charlson‐Deyo score. Predictors of OS were evaluated via a univariate and multivariate Cox proportional hazard model. SPSS Statistics Version 28 (IBM Corp., Armonk, N.Y., United States) was used for all analyses, with a two‐tailed alpha of 0.05 indicating significance.

## 3 RESULTS

Of 542 R‐NEN cases, 166 (31%) were NET‐G1, 14 (31%) were NET‐G2, 169 (3%) were NEC‐NOS, 18 (3%) were LC‐NEC and 175 (32%) were SC‐NEC (Table 1). The median age at diagnosis was 62. Sixty‐nine per cent of these tumours were treated in academic centres or comprehensive community cancer programmes. Thirty‐four per cent of the NEN in this study were Stage 4 at diagnosis. Treatment options for patients included surgery (61%), chemotherapy (30%), radiation (13%) and immunotherapy (2%). Of the patients who underwent surgery, 73% underwent radical nephrectomy compared to 23% who underwent nephron sparing surgery and 4% who underwent local excision or ablation. Most of the surgical cases were performed through minimally invasive techniques with 25% conducted laparoscopically and 29% robotically compared to 46% open.

**TABLE 1:** Baseline characteristics of renal neuroendocrine neoplasms in NCDB

			*n* = 542
Age at diagnosis [IQR]			62 [50–73]
Age groups, *n* (%)			
	<50		131 (24.2)
	≥50		411 (75.8)
Gender, *n* (%)			
	Male		281 (51.8)
	Female		261 (48.2)
Race, *n* (%)			
	White		250 (83.0)
	Black		58 (10.7)
	American Indian		2 (0.4)
	Asian and Pacific Islander		22 (4.1)
	Other		4 (0.7)
	Unknown		6 (1.1)
Hispanic, *n* (%)			
	Hispanic		25 (4.6)
	Not Hispanic		486 (89.7)
	Unknown		31 (5.7)
Insurance, *n* (%)			
	Private insurance/managed care		241 (44.5)
	Not insured		13 (2.4)
	Medicaid		36 (6.6)
	Medicare		234 (43.2)
	Other government		4 (0.7)
	Unknown		14 (2.6)
Rural/urban, *n* (%)			
	Rural		8 (1.5)
	Urban		509 (93.9)
	Unknown		25 (4.6)
Facility type, *n* (%)			
	Academic/research program		187 (34.5)
	Comprehensive community cancer program		187 (34.5)
	Community cancer program		31 (5.7)
	Integrated network cancer program		81 (14.9)
	Unknown		56 (10.3)
Charlson–Deyo score, *n* (%)			
	0		406 (74.9)
	1		89 (16.4)
	2		35 (6.5)
	≥3		12 (2.2)
Histology, *n* (%)			
	NET, Grade 1 (NET‐G1)		166 (30.6)
	NET, Grade 2 (NET‐G2)		14 (2.6)
	NEC, NOS (NEC‐NOS)		169 (31.2)
	Large cell NEC (LC‐NEC)		18 (3.3)
	Small cell NEC (SC‐NEC)		175 (32.3)
Tumour size, *n* (%)			
	≤ 2 cm		357 (65.9)
	>2 and <4 cm		7 (1.3)
	>4 cm		2 (0.4)
	Unknown/undocumented		176 (32.5)
Laterality, *n* (%)			
	Left		253 (6.7)
	Right		282 (52.0)
	Bilateral/other/unknown		7 (1.3)
AJCC T stage at diagnosis, *n* (%)			
	T1		119 (22.0)
	T2		66 (12.2)
	T3		68 (12.5)
	T4		37 (6.8)
	TX/unknown		252 (46.5)
AJCC N stage at diagnosis, *n* (%)			
	N0		203 (37.5)
	N1		107 (19.7)
	N2		13 (2.4)
	NX/unknown		219 (40.4)
AJCC M stage at diagnosis, *n* (%)			
	M0		247 (45.6)
	M1		160 (29.5)
	MX/unknown		135 (24.9)
Sites of metastasis (if known), *n* (%)			
	Bone		20/160 (12.5)
	Brain		8/160 (5.0)
	Liver		30/160 (18.8)
	Lung		20/160 (12.5)
AJCC clinical stage at diagnosis, *n* (%)			
	Stage 1		75 (13.8)
	Stage 2		27 (5.0)
	Stage 3		58 (10.7)
	Stage 4		185 (34.1)
	Other/unknown		197 (36.3)
Chemotherapy, *n* (%)			
	Yes		160 (29.5)
		Adjuvant	47/160 (29.4)
		Neoadjuvant	5/160 (3.1)
		Sequence unknown	8/160 (5.0)
		No associated surgery	100/160 (62.5)
	None		335 (61.8)
	Unknown		47 (8.7)
Immunotherapy, *n* (%)			
	Yes		8 (1.5)
	No		534 (98.5)
Radiation therapy, *n* (%)			
	Yes		71 (13.1)
		Adjuvant	21/71 (29.6)
		Neoadjuvant	0/71 (0.0)
		Sequence unknown	14/71 (19.7)
		No associated surgery	36/71 (50.7)
	No		471 (86.9)
Surgery, *n* (%)			
	Yes		328 (60.5)
	No		214 (39.5)
Surgery rates by stage, *n* (%)			
	T1‐T2, non‐metastatic		107/122 (87.7)
	T3‐T4, non‐metastatic		43/59 (72.9)
	Metastatic		37/160 (23.1)
Type of surgery, *n* (%)			
	Local excision/ablation		12 (3.7)
	Partial nephrectomy		76 (23.2)
	Nephrectomy		239 (73.1)
Surgical approach, *n* (%)			
	Open		92 (45.8)
	Laparoscopic		51 (25.4)
	Robotic		58 (28.9)
Regional lymph node dissection, *n* (%)			
	Yes		129 (23.8)
	No		405 (74.7)
	Unknown		8 (1.5)
Median days from diagnosis to surgery [IQR]			14 [0–43]
Surgical margins, *n* (%)			
	Negative		248 (45.8)
	Positive		55 (10.1)
	Not applicable/unknown		239 (44.1)

*Note*: Categorical variables reported using proportions and continuous variables reported using medians and interquartile ranges.

**FIGURE 1:**
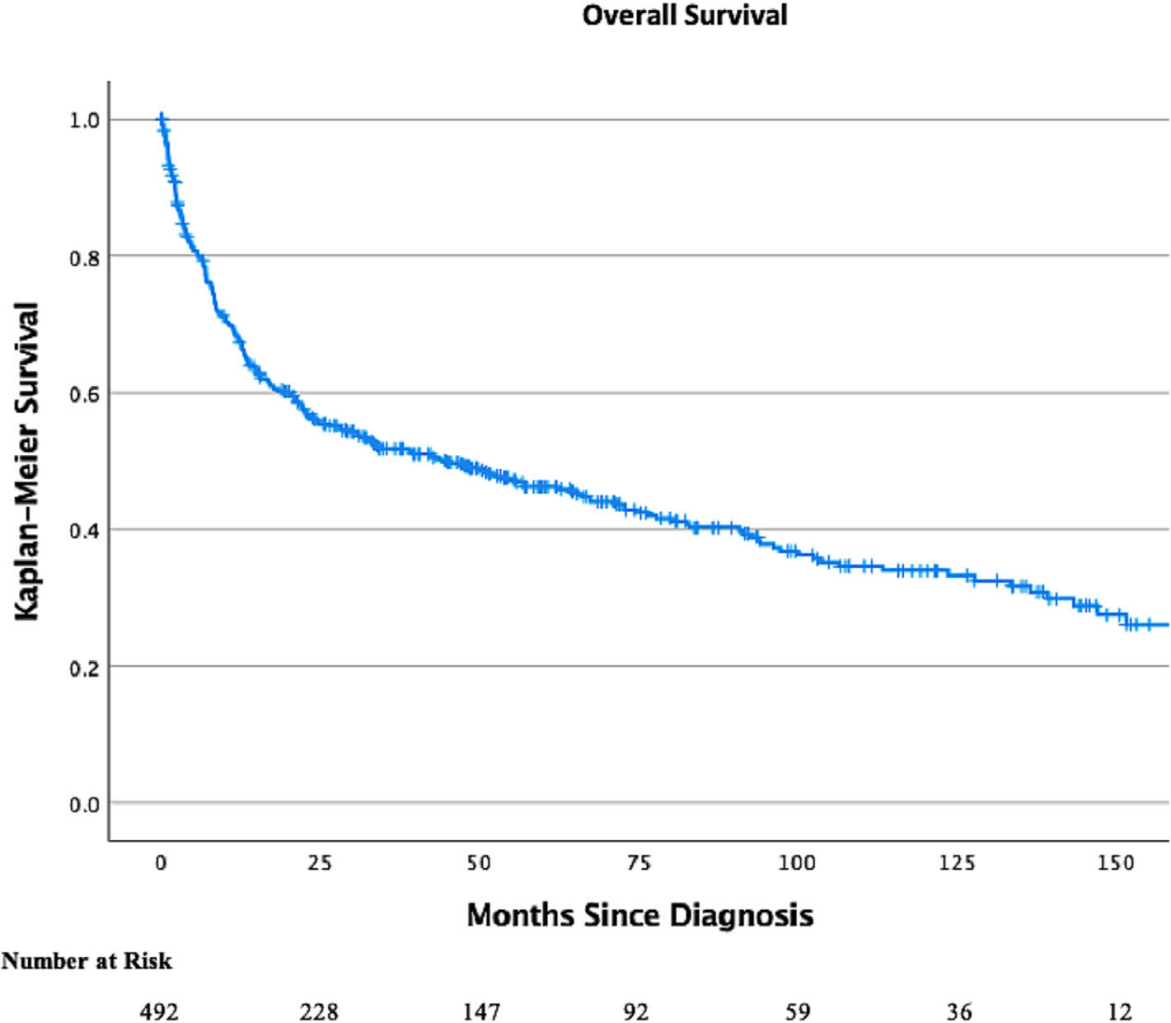
Kaplan Meier curve on overall survival in patients with R‐NEN. Notches on lines indicate censored cases.

**FIGURE 2:**
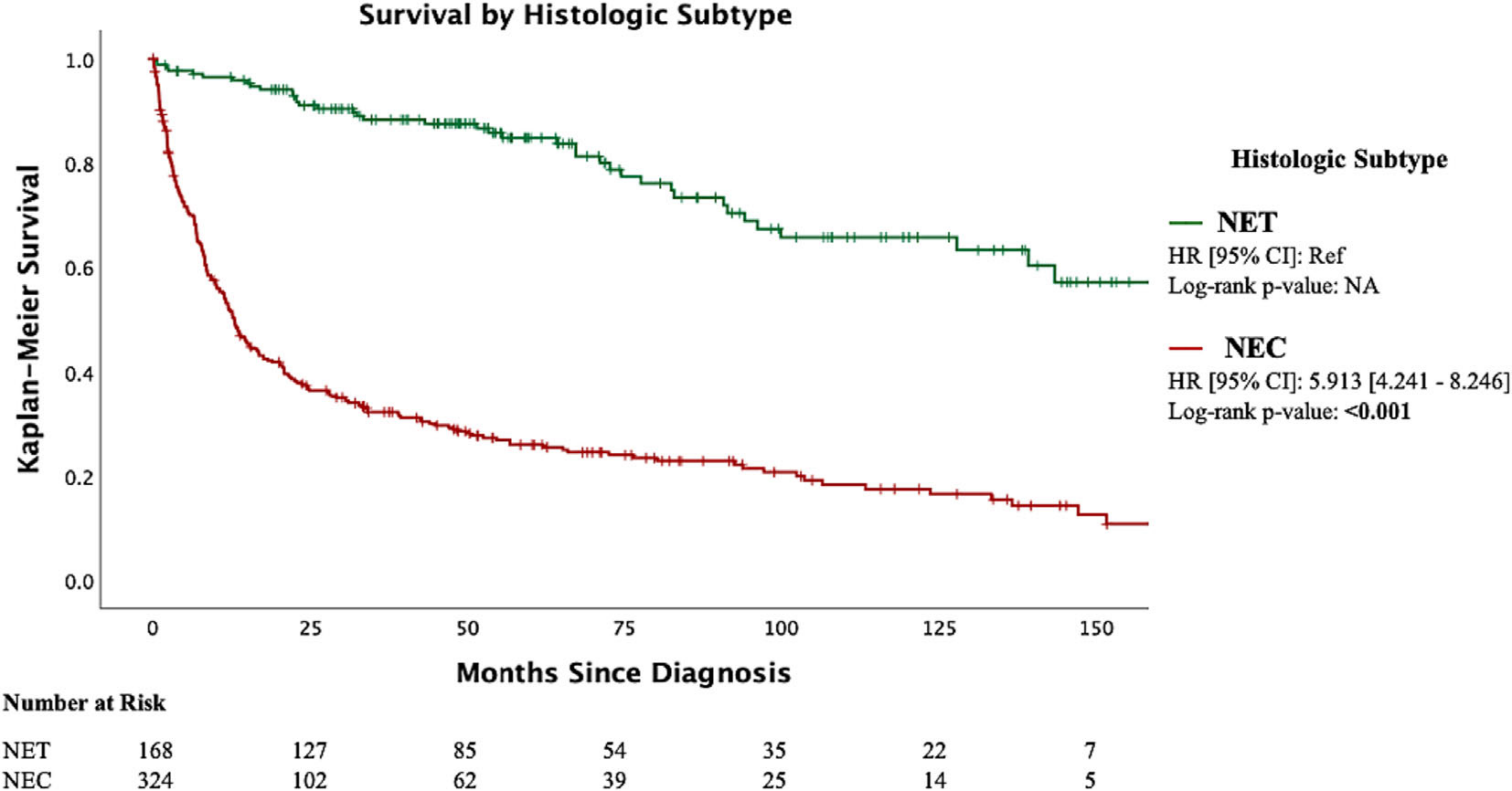
Kaplan Meier curve on overall survival in patients with R‐NEN separated by histologic subtype: NET versus NEC. NET was defined as Grade 1 or 2 neuroendocrine tumours; NEC was defined as large cell, small cell or NOS neuroendocrine carcinoma. Notches on lines indicate censored cases.

**FIGURE 3:**
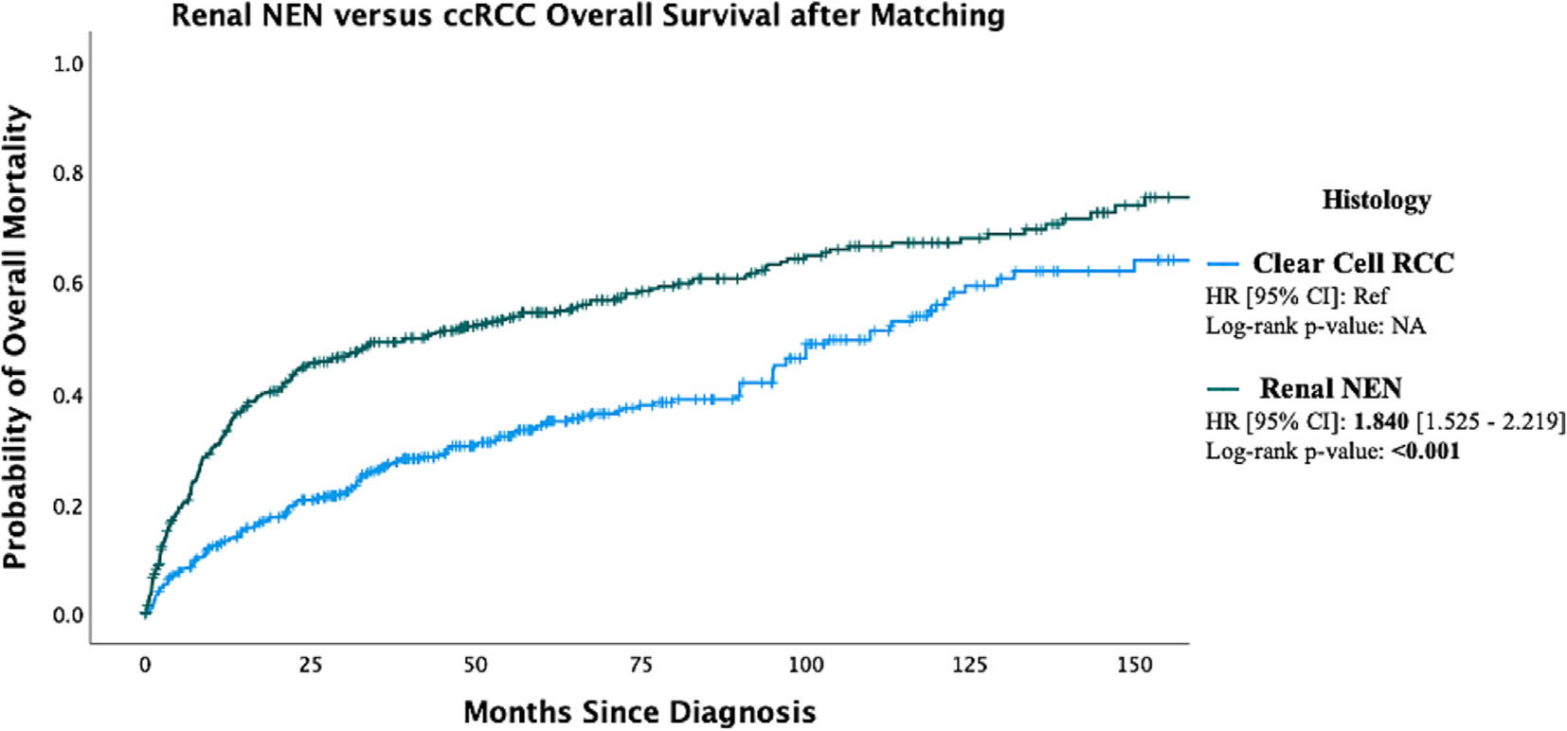
Kaplan Meier curve showing probability of overall mortality in patients with R‐NEN versus clear cell RCC after matching. Cases matched on age, sex, AJCC T stage and Charlson–Deyo score. Notches on lines indicate censored cases.

**FIGURE 4:**
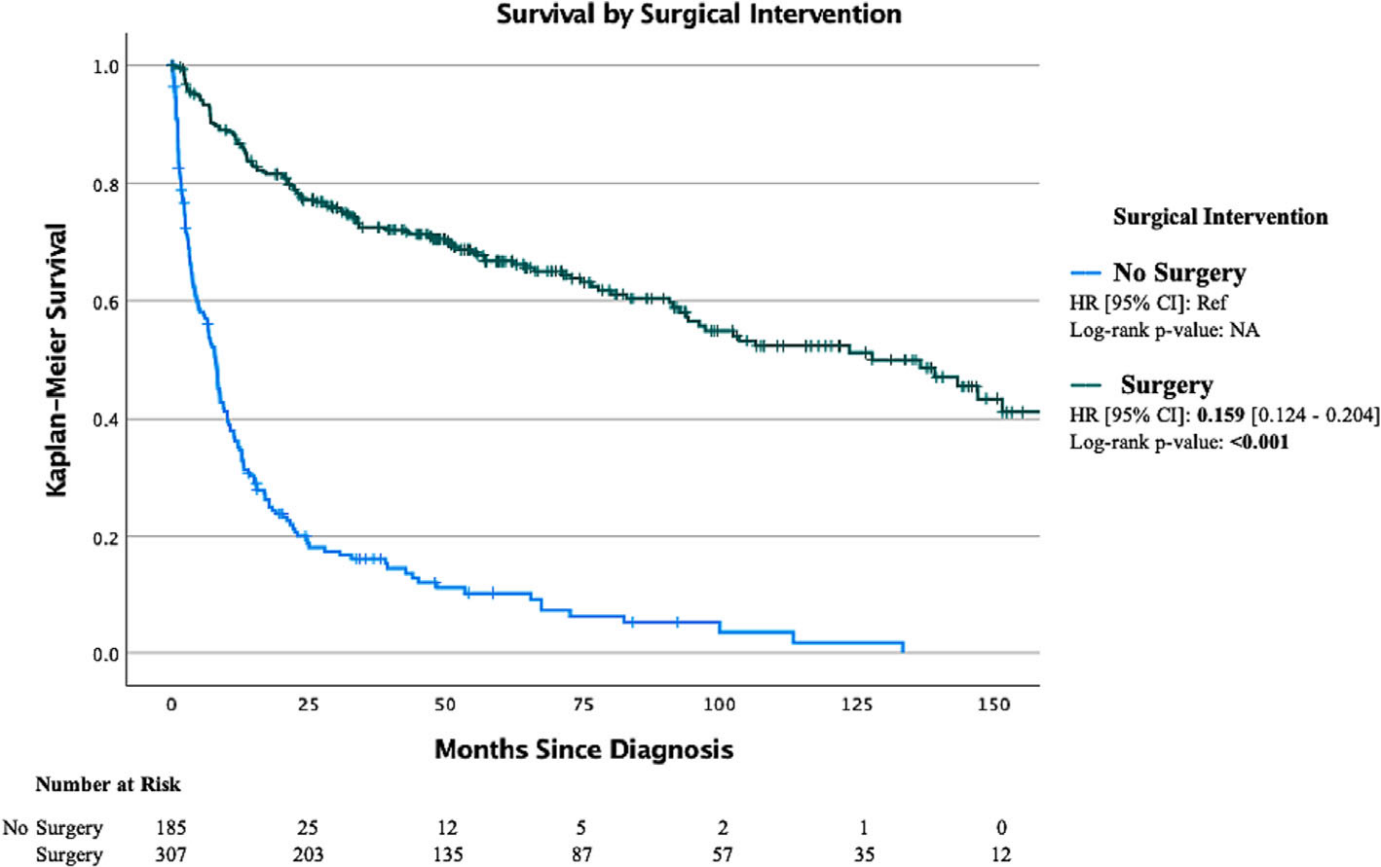
Kaplan Meier curve on overall survival for patients based on surgical intervention. Hazard ratio (HR) is for overall mortality. Notches on line indicate censored data.

Median overall survival for all patients in the study was 44.9 months (SE, 4.265; 95% CI, 27.6–62.2) (Figure 1). Patients with a NEC histologic subtype (LC‐NEC, SC‐NEC or NOS‐NEC) were at a significantly increased risk of mortality (HR, 5.91; 95% CI, 4.24–8.25; *p* < 0.001) when compared to those with NET (NET‐G1 or NET‐G2) neoplasms (Figure 2). Patients diagnosed with R‐NEN are at an increased risk of all‐cause mortality (HR, 1.84; 95% CI, 1.53–2.22; *p* < 0.001) when compared to those diagnosed with ccRCC once matched on age, sex, AJCC T stage and Charlson–Deyo score (Figure 3). The median overall survival was 139.20 months (SE, 22.5; 95% CI, 95.22–183.23) for clinical stage I disease, 56.9 months (SE, 22.96; 95% CI, 11.98–101.94, *p* < 0.001) for clinical stage III disease and 7.89 months (SE 0.62; 95% CI, 6.68–9.10, *p* < 0.001) for clinical stage IV disease (Figure S1). The median overall survival was not reached for clinical stage II disease. The median overall survival was 94.19 (SE, 16.80; 95% CI, 61.25–127.13) for pathologic T1 disease, 54.60 months for T2 disease, 21.68 months (SE, 4.44; 95% CI, 12.98–30.39, *p* = 0.014) for T3 disease and 4.63 months (SE, 1.02; 95% CI, 2.63–6.63, *p* < 0.001) for T4 disease. Median overall survival was 7.89 months (SE 0.67; 95% CI, 6.58–9.20) for patients without surgical intervention and 136.61 months (SE 16.44; 95% CI, 104.38–168.84, *p* < 0.001) for patients who underwent surgery (Figure 4).

Increased age (HR, 1.05; 95% CI, 1.03–1.06; *p* < 0.001), T4 stage disease (HR, 3.17; 95% CI, 1.96–5.1; *p* < 0.001), NEC‐NOS histology (HR, 2.82; 95% CI, 1.64–4.86; *p* < 0.001), LC‐NEC histology (HR, 2.73; 95% CI, 1.04–7.17; *p* = 0.041) and SC‐NEC histology (HR, 5.17; 95% CI, 2.95–9.05; *p* < 0.001) were all positive predictors of worsening overall survival (Table 2).

**TABLE 2:** Univariate and multivariate cox regression survival analysis of NET patients

Factor	Univariate			Multivariate		
	HR	95% CI	*P* value	HR	95% CI	*P* value
Age at diagnosis	1.058	1.048–1.067	<0.001*	1.046	1.033–1.060	<0.001*
Gender						
Male	Ref	‐	‐	Ref	‐	‐
Female	0.630	0.498–0.797	<0.001*	0.838	0.608–1.156	0.282
Charlson–Deyo Score						
0	Ref	‐	‐	Ref	‐	‐
1	1.713	1.285–2.285	<0.001*	1.123	0.748–1.687	0.575
≥2	1.512	0.944–2.421	0.085	0.646	0.342–1.219	0.177
T Stage						
1	Ref	‐	**‐**	Ref	‐	‐
2	0.976	0.630–1.514	0.915	0.858	0.542–1.357	0.513
3	1.616	1.092–2.393	0.016*	1.052	0.685–1.617	0.816
4	5.031	3.231–7.834	<0.001*	3.174	1.959–5.144	<0.001*
Surgical margins						
Negative	Ref	‐	**‐**	Ref	‐	**‐**
Positive	3.179	2.157–4.684	<0.001*	0.690	0.428–1.113	0.128
Histology						
Grade 1 or 2 neuroendocrine tumour	Ref	‐	‐	Ref	‐	‐
Neuroendocrine carcinoma, NOS	4.128	2.870–5.935	<0.001*	2.823	1.638–4.864	<0.001*
Large cell neuroendocrine carcinoma	6.483	3.466–12.126	<0.001*	2.732	1.042–7.165	0.041*
Small cell neuroendocrine carcinoma	9.198	6.429–13.140	<0.001*	5.165	2.947–9.051	<0.001*

*Note*: Showing univariate and multivariate Cox regression survival analysis of NET patients. Significance set to *p* < 0.05 (bolded and * for reference).

Abbreviations: HR, hazard ratio; Ref, reference.

## 4 DISCUSSION

Studies have shown an increased incidence and prevalence of NEN in the last decade, yet primary R‐NEN remains a poorly understood subset due to its rarity.^1^ This study represents the largest population‐based investigation of this rare tumour. Our study shows that a majority of these tumours are treated in academic centres or comprehensive community cancer programmes. Most of these tumours are treated with surgery though 30% of patients underwent chemotherapy, and 13% had radiation. The median overall survival for patients in our series with R‐NEN was 44 months. There was a significant difference in overall survival between patients who underwent surgery and those who pursued more conservative treatments (136.61 months vs. 7.89 months). Increased age, higher stage, NEC‐NOS histology, LC‐histology and SC‐NEC histology were all predictors of worsening overall survival.

The origin of R‐NEN is still unclear, as there are no known neuroendocrine cells within the renal parenchyma.^3^ Studies suggest that R‐NEN precursor cells arise from renal stem cells that develop towards neuroendocrine differentiation.^12^ Previous studies suggest that metastasis is common at the time of diagnosis, typically to the liver or regional lymph nodes, even in patients with well‐differentiated tumours.^4^, ^8^ There are a few associated conditions with R‐NEN, specifically horseshoe kidney and renal teratomas.^13^, ^14^, ^15^ R‐NENs often share features found in others NENs including immunohistochemical expression of chromogranin A and synaptophysin. Yet studies have shown that they have highly variable mutational profiles and their oncologic potential can be quite heterogenous, making it difficult to predict outcomes based on histology alone.^11^ Overall, studies have confirmed that well‐differentiated R‐NENs tend to have better outcomes than those with poorly differentiated features, but information on survival outcomes based on histological subtype is still lacking.^3^

There have been no clinical trials establishing treatment guidelines for R‐NENs. Current treatment options are extrapolated from renal cancers and neuroendocrine cancers alike. First‐line treatment for localized R‐NEN is often nephrectomy with lymph node dissection.^16^ For those with metastatic R‐NENs, systemic treatments used for other neuroendocrine tumours such as peptide‐receptor radio nucleotide therapy, tyrosine kinase inhibitors, somatostatin analogues and chemotherapy are often considered.^16^, ^17^ In the largest single‐institution based study on R‐NEN, McGarrah et al.^8^ assessed 17 patients with primary R‐NEN with a median follow‐up 62.8 months. Of the 17 patients, 16 underwent surgical resection with 13 of these also undergoing lymph node dissection at time of surgery. Lymph node positive disease was identified in 92% of these patients. They reported a median overall survival of 143 months. The difference in overall survival between their study and the current one may be attributed to different patient populations as more than 53% of their patients had well‐differentiated tumours compared to only 16% in our study. As stated above, most studies have shown that well‐differentiated NETs are relatively indolent.^18^

As this is the largest population‐based study on R‐NEN, it is important to compare our findings to the current literature. Nguyen et al.^17^ assessed the Surveillance, Epidemiology and Results (SEER) database over a four‐decade period to identify 166 cases of R‐NEN. They reported a 5‐year overall survival of 50%. Similar to the current study, 34% of patients in Nguyen's study had NET‐G1 tumours compared to 30% in the present study. The Nguyen study conducted a sub‐analysis of removing patients with SC‐NEC in their median overall survival calculation and noted that their median overall survival rose to 8.9 years. The authors note that the breakdown of histology, specifically in regards to a paucity of NET‐G2 and LC‐NEC, meant that the survival data they published was likely underpowered. Their study additionally confirmed the results in the present study by showing that increased mortality was associated with older age, regional and distant disease, and SC‐NEC histology.

Our study is not without limitations. We recognize the limitations associated with a retrospective study design. The lack of granularity in the data did not allow us to understand clinically relevant end‐points such as complications, cause‐of‐death, local control and disease‐free survival. Additionally, considering one of the major conclusions of most neuroendocrine studies is the association between certain histologic subtypes and worsening disease, the retrospective design did not allow for centralized review of pathologic specimens. Finally, though the NCDB includes data on staging, there is no official American Joint Committee on Cancer staging system for R‐NENs, highlighting the importance of randomized controlled trials to better characterize these tumours.

Our study is with several strengths, the greatest of which is that this is the largest hospital‐based analysis on R‐NEN in the literature. The data published here, though not generalizable, can expose some of the nuances of this rare tumour by leveraging the large study cohort. The NCDB has been cited throughout the literature for its internal validity as a joint quality improvement program of the Commission on Cancer (CoC) between the American College of Surgeons and the American Cancer Society.^19^ Ultimately, this study can serve as a catalyst for prospective studies and clinical trials to better understand and characterize treatment options and response for R‐NEN.

## 5 CONCLUSION

R‐NEN is an aggressive tumour characterized by high mortality rates, particularly for patients with NEC‐NOS and SC‐NEC histology. Surgery continues to be the mainstay of treatment and has shown to provide a survival benefit, but further studies are needed to help improve outcomes for these patients.

## AUTHOR CONTRIBUTIONS

All authors were involved in project design. Dr. Omidele and Dr. Connors were involved in data analysis and writing.

## CONFLICT OF INTEREST

The authors declare no conflict of interest.

## Supporting information


**Figure S1:** Kaplan Meier curve on overall survival for patients based on (a) AJCC clinical stage (b) AJCC T stage. Hazard ratio (HR) is for overall mortality. Notches on line indicate censored data.
