# Supplementary material for: Prognostic factors and treatment impact on overall survival in patients with renal neuroendocrine tumour
Source: BJUI Compass. 2024 Mar 11;5(6):576–84. doi: 10.1002/bco2.341 (PMC11168774; doi:10.1002/bco2.341)
Supplement: Supplementary file 1 — Figure S1: Kaplan Meier curve on overall survival for patients based on (a) AJCC clinical stage (b) AJCC T stage. Hazard ratio (HR) is for overall mortality. Notches on line indicate censored data. [file BCO2-5-576-s001.docx]

**Supplementary Figures:**

**
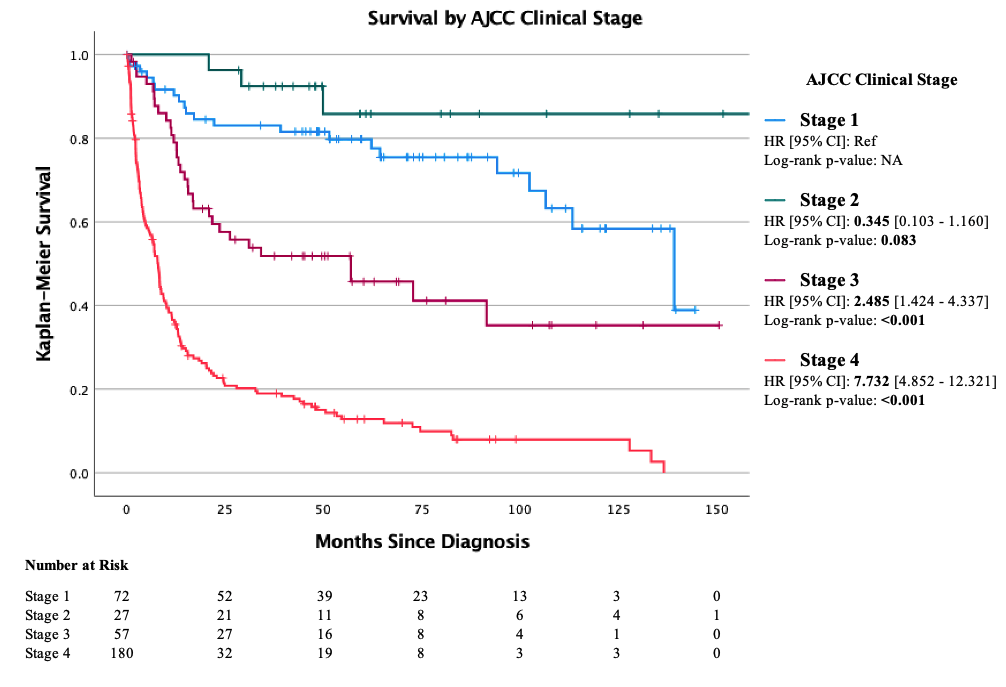
**

**
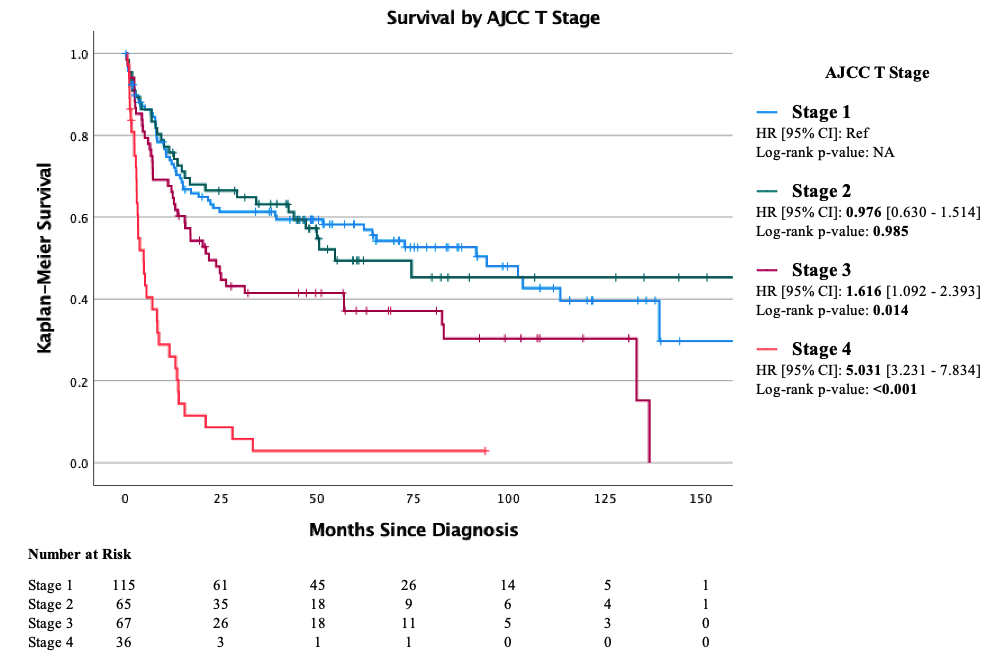
**

Supplementary 1: Kaplan Meier curve on overall survival for patients based on (a) AJCC clinical stage (b) AJCC T stage. Hazard ratio (HR) is for overall mortality. Notches on line indicate censored data.
